# Sleep disturbances, depressive symptoms, and cognitive efficiency as determinants of mistakes at work in shift and non-shift workers

**DOI:** 10.3389/fpubh.2022.1030710

**Published:** 2022-12-14

**Authors:** Hyewon Yeo, Jooyoung Lee, Sehyun Jeon, Somi Lee, Yunjee Hwang, Jichul Kim, Seog Ju Kim

**Affiliations:** ^1^Department of Psychiatry, Samsung Medical Center, Sungkyunkwan University College of Medicine, Seoul, South Korea; ^2^Department of Brain and Cognitive Engineering, Korea University, Seoul, South Korea

**Keywords:** shift work, depression, sleep, cognition, performance, multi-group SEM

## Abstract

**Introduction:**

Shift work is known to reduce productivity and safety at work. Previous studies have suggested that a variety of interrelated factors, such as mood, cognition, and sleep, can affect the performance of shift workers. This study aimed to identify potential pathways from depression, sleep, and cognition to work performance in shift and non-shift workers.

**Material and methods:**

Online survey including the Center for Epidemiologic Studies Depression Scale (CES-D), Cognitive Failure Questionnaire (CFQ), and Pittsburgh Sleep Quality Index (PSQI), as well as two items representing work mistakes were administered to 4,561 shift workers and 2,093 non-shift workers. A multi-group structural equation model (SEM) was used to explore differences in the paths to work mistakes between shift and non-shift workers.

**Results:**

Shift workers had higher PSQI, CES-D, and CFQ scores, and made more mistakes at work than non-shift workers. The SEM revealed that PSQI, CES-D, and CFQ scores were significantly related to mistakes at work, with the CFQ being a mediating variable. There were significant differences in the path coefficients of the PSQI and CES-D between shift and non-shift workers. The direct effects of sleep disturbances on mistakes at work were greater in shift workers, while direct effects of depressive symptoms were found only in non-shift workers.

**Discussion:**

The present study found that shift workers made more mistakes at work than non-shift workers, probably because of depressed mood, poor sleep quality, and cognitive inefficiency. Sleep influences work performance in shift workers more directly compared to non-shift workers.

## Introduction

Shift work is typically referred to as an employment practice designed to provide all-day services (1). Globally, the number of people engaged in shift work has been rapidly increasing to meet the demand for 24-h service. Nowadays, shift work became common in most countries, with 10–40% of workers engaged in shift work (2).

With the increasing importance of shift work in the modern era, the high performance of shift workers is important for efficiency and safety at work. However, successive night shifts decrease safety and lead to mistakes at work (3). Night-shift work is associated with difficulties in performing routine tasks, poor performance, and increased rates of accidents and injuries (4).

Irregular sleep patterns of shift workers may reduce work efficiency. Shift workers are known to experience significant difficulty in initiating and maintaining sleep (5) because their work schedule conflicts with the natural biological clock (6). Disturbances in the circadian rhythm affect not only the sleep-wake cycle but also sleep quality and duration (7). Sleep problems, such as insomnia, and obstructive sleep apnea, impair productivity at work (8).

Cognitive deficits associated with shift work may reduce work efficiency. A laboratory study demonstrated that circadian misalignment in shift workers decreases subjective alertness and the ability to sustain attention, cognitive throughput, information processing, and visuomotor performance (9). As cognitive functions are required to concentrate on goals, plan strategies, and organize tasks, even subtle cognitive impairment can influence the performance of a broad range of tasks at work (10).

Depressed mood may also influence job performance in shift workers. Several studies have reported that shift workers experience a wide range of mental health problems. In particular, the risk of depression was found to be higher in shift workers (11). Impaired performance was not just limited to clinical depression patients but was also present in workers with subclinical depressive symptoms (12).

Previous studies have demonstrated a close relationship between sleep disturbances, cognitive efficiency, and depressive symptoms. Insomnia or hypersomnia and diminished ability to think or concentrate are core diagnostic criteria for major depressive disorder (13). Poor sleep quality and duration cause a broad range of cognitive impairments, including in attention, memory, and executive function (14). Sleep disturbances and depressive symptoms appear to influence each other (15, 16). Although the effect of each of these three variables on performance at work is well-established, the underlying mechanisms remain unclear.

This study aimed to investigate the potential effects of depression, sleep, and cognition on the performance of shift and non-shift workers. On the basis of previous studies, we formulated the following hypotheses. First, there would be a difference in depressive symptoms, sleep disturbances, cognitive efficiency, and mistakes at work between shift and non-shift workers. Second, there would be an indirect effect of depressive symptoms and sleep disturbance on mistakes at work through cognitive efficiency. Finally, the pathways from depressive symptoms, sleep disturbances, and cognitive efficiency to mistakes at work would be different between shift and non-shift workers.

## Materials and methods

### Study participants

A total of 6,665 participants were recruited, of whom 11 were excluded because their work type was difficult to classify. The remaining 6,654 participants (4,561 shift workers and 2,093 non-shift workers) completed all assessments and were thus included in the final analysis. Initially, 1,254 participants (448 males and 806 females; 961 shift and 293 non-shift workers) were recruited *via* an online advertisement. The majority of the respondents to the online advertisement were young female shift workers; an online survey company (Macromill Embrain Co., Ltd., South Korea) was employed to recruit an additional 5,400 participants (2,693 males and 2,707 females; 3,600 shift workers and 1,800 non-shift workers), especially males, middle-aged workers, and non-shift workers.

Adult participants (aged > 18 years) in full- or part-time employment were included, and only those who could not complete the online survey were excluded. All procedures were performed in accordance with the ethical standards of the relevant institutional committees on human experimentation and the Declaration of Helsinki (2013). The study protocol was approved by the Institutional Review Board of Samsung Medical Center (Protocol Code: 2019-04-095). Informed consent was obtained from all participants after an explanation of the survey.

### Data collection

All data were collected *via* the online survey among the general population of the Republic of Korea, from 2019 to 2021. Depressive symptoms were assessed using the Korean version of the short-form of the Center for Epidemiologic Studies Depression Scale (CES-D) (17–19). The short-form K-CES-D is a self-reported questionnaire with scores ranging from 0 to 33; higher scores indicate more severe depressive symptoms. The cut-off score of the short-form K-CES-D is 16 for depression screening in the Korean population (20).

Sleep disturbance was assessed using the Korean version of the Pittsburgh Sleep Quality Index (K-PSQI) (21, 22). The K-PSQI is a self-reported questionnaire consisting of 19 items that assess seven dimensions of sleep over 1 month. These include subjective quality, latency, duration, efficiency, disturbance, use of sleep medication, and daytime dysfunction. The total PSQI score is calculated by summing the scores of all dimensions, and ranges from 0 to 21. A higher score indicates worse sleep quality. The cut-off score of K-PSQI is 8.5 to evaluate sleep disorders in the Korean population (22).

Cognitive efficiency was assessed using the Korean version of the Cognitive Failure Questionnaire (CFQ) (23, 24). The CFQ is a self-reported questionnaire that assesses failures of memory, action, and perception in everyday life. It consists of 25 self-rated items; scores range between 0 and 100, with higher scores indicating greater cognitive impairment.

Mistakes at work were assessed using two items evaluating the mistakes at work that respondents had ever made. One item pertained to actual mistakes, including minor ones, while the other was concerned with “near-mistakes”. The frequency of mistakes and near-mistakes was graded from 1 (never) to 6 (>3 per month) for each item.

### Statistical analysis

Differences of demographic characteristics between shift and non-shift workers were assessed using the *t*-test or chi-square test. Then, differences of clinical characteristics between the two groups using analysis of covariance (ANCOVA) after controlling for age and sex. Spearman correlation analyses of depressive symptoms, cognitive efficiency, sleep disturbance, and mistakes in performance were also performed. The strength of the Spearman correlation is interpreted with 0.00–0.29 as weak, 0.30–0.59 as moderate, and 0.60–1.00 as strong (25).

In accordance with the guidelines of Morrison et al. (26), a multi-group structural equation model (SEM) was constructed using IBM SPSS AMOS (version 26.0; IBM Corp.). We performed a confirmatory factor analysis (CFA) to examine a priori the interrelationships that are theorized to exist. In this step, the properties of the scales were assessed to determine whether the measurement model had an acceptable fit to the data. Regarding mistakes at work, an explanatory factor analysis was performed to derive a one-factor model. All models are described in Supplementary material.

**Figure 1:**
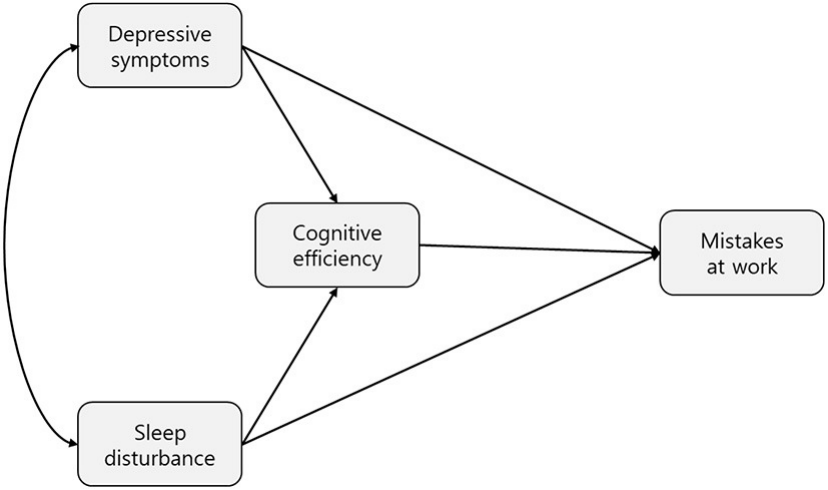
Hypothetical model of the effects of depressive symptoms, sleep disturbances, and cognitive efficiency on mistakes at work. The hypothetical model includes four variables: depressive symptoms, sleep disturbances (predictor variables), mistakes at work (response variable), and cognitive efficiency (mediator variable).

Although the relationships among depression, sleep, and cognition in shift workers are well-known, a theoretical model for shift workers is not yet established. Our structural model was based on one of the theoretical, evidence-based models for the functioning of bipolar affective disorder (27). Our model included three latent variables, i.e., cognitive efficiency, sleep disturbances, and depressive symptoms, and one observed variable, i.e., the mean score of the two items on mistakes at work (Figure 1). The data fit of the individual models and overall (multi-group) model was computed separately as well as the overall multi-group model. We evaluated the model fit using the chi-squared statistic with normed chi-square (χ^2^/df), root mean square error of approximation (RMSEA), and comparative fit index (CFI). To assess group differences in the magnitude of paths between shift and non-shift workers, the chi-square-difference test was performed to determine whether a given scale or test had equivalent measurement properties in groups. All data were analyzed using SPSS Statistics (version 27.0; IBM Corp., Armonk, NY, USA). All tests were two-sided, and a *p*-value < 0.05 was considered statistically significant.

## Results

### Sample characteristics

A total of 6,654 participants, including 4,561 shift workers and 2,093 non-shift workers, were enrolled in this study. The mean age was 37.5 years and 47.2% (*n* = 3,141) were males. About half of the participants were married (52.2%). The mean working years and working hours per week were 11.4 years and 30.5 h, respectively.

### Group differences in demographical and clinical characteristics

There were no group differences in age and sex. Shift workers had worked for fewer years (*p* < 0.001) and had longer working hours per week (*p* < 0.001) compared to non-shift workers. The proportion of unmarried individuals was higher in the shift workers group (*p* = 0.035) (Table 1).

**Table 1:** Demographic characteristics of the study participants (*N* = 6,654).

	**Shift**	**Non-shift**	
	**(*n =* 4,651)**	**(*n =* 2,093)**	***P*-value**
	M ± SD or *n* (%)	M ± SD or *n* (%)	
Age (years)	37.4 ± 28.2	37.8 ± 9.7	0.561
**Sex**
Male	2,142 (47.0)	999 (47.7)	0.517
Marital status			0.035
Married	2,022 (44.3)	989 (47.3)	
Single	2,418 (53.0)	1,056 (50.5)	
Divorced/bereaved	121 (2.7)	48 (2.3)	
**Employment**
Years of employment	11.1 ± 8.6	12.0 ± 8.6	< 0.001
Hours of work per week	31.7 ± 20.8	28.2 ± 18.2	< 0.001
Monthly income (thousand won)			< 0.001
under 1,500	300 (6.6)	72 (3.4)	
1,500 ~ 2,500	546 (12.0)	347 (16.6)	
2,500 ~ 3,500	1,973 (43.3)	920 (44.0)	
3,500 ~ 4,500	1,334 (29.2)	522 (24.9)	
over 4,500	408 (8.9)	232 (11.1)	

M, mean; SD, standard deviation.

Compared to non-shift workers, shift workers had higher PSQI (7.1 ± 3.60 vs. 6.3 ± 3.23, *F* = 35.699, *p* < 0.001), CES-D (8.8 ± 6.24 vs. 7.1 ± 5.84, *F* = 21.447, *p* < 0.001), and CFQ (27.3 ± 18.7 vs. 24.7 ± 17.29, *F* = 22.375, *p* < 0.001) scores after adjusting for age and sex (Table 2). In addition, the score assessing mistakes at work was also higher for shift workers (3.5 ± 2.06) than non-shift workers (3.0 ± 1.83, *F* = 54.733, *p* < 0.001). 45.04% of shift workers had sleep disorders, compared with 33.20% of non-shift workers. Moderate to severe sleep disturbances were found in 44.16 and 30.86% of the shift and non-shift workers, respectively. On the short-form K-CES-D, 15.13 and 9.79% of the shift and non-shift workers were identified with clinical depression, respectively.

**Table 2:** Clinical characteristics of shift and non-shift workers.

	**Shift workers**	**Non-shift workers**	**F-statistic**	***P*-value**
	***M*** **(SD)**	***M*** **(SD)**		
PSQI	7.1 (3.60)	6.3 (3.23)	35.699	< 0.001
CES-D	8.8 (6.24)	7.1 (5.84)	21.447	< 0.001
CFQ	27.3 (18.7)	24.7 (17.29)	22.375	< 0.001
Mistakes at work	3.5 (2.06)	3.0 (1.83)	54.733	< 0.001

Model adjusted for age and sex. The cut-off score of CES-D is 16 for depression. The cut-off score of PSQI is 8.5 for sleep disorders.

PSQI, Pittsburg Sleep Quality Index; CES-D, Center for Epidemiologic Studies Depression Scale; CFQ, Cognitive Failure Questionnaire; M, mean; SD, standard deviation.

### Relationships among depressive symptoms, sleep disturbance, cognitive efficiency, and mistakes at work

All of the variables were statistically significantly correlated with each other (*p* < 0.01). Mistakes at work was moderately associated with sleep disturbances (*r* = 0.308), cognitive efficiency (*r* = 0.358), and depressive symptoms (*r* = 0.353). Cognitive efficiency was strongly correlated with depressive symptoms (*r* = 0.548) and was moderately correlated with sleep disturbances (*r* = 0.342). Sleep disturbances and depressive symptoms were also strongly correlated (*r* = 0.509) (Supplementary Table S1).

Similar results were found in both groups (*p* < 0.01). For the shift-workers group, mistakes at work was moderately associated with sleep disturbances (*r* = 0.308), cognitive efficiency (*r* = 0.370), and depressive symptoms (*r* = 0.347). Cognitive efficiency was strongly associated with depressive symptoms (*r* = 0.561) and moderately associated with sleep disturbances (*r* = 0.351). Sleep disturbances and depressive symptoms were strongly correlated (*r* = 0.517). For the non-shift workers group, mistakes at work was moderately associated with cognitive efficiency (*r* = 0.320), and depressive symptoms (*r* = 0.329), but was weakly associated with sleep disturbances (*r* = 0.260). Cognitive efficiency was strongly associated with depressive symptoms (*r* = 0.511) and moderately associated with sleep disturbances (*r* = 0.306). Sleep disturbances and depressive symptoms were strongly correlated (*r* = 0.457).

### Structural equation model of depressive symptoms, sleep disturbances, cognitive efficiency, and mistakes at work

The hypothesized structural model was tested, and the standardized path coefficients are presented in Supplementary Table S2. The overall multi-group model fit the data well [χ^2^ (df = 114, *N* = 6,665) = 3,874.272, *p* < 0.001, CFI = 0.935, TLI = 0.912, RMSEA = 0.070, 95% CI RMSEA (0.068, 0.072)], and all structural paths were significant at *p* < 0.001. All path coefficients were significant in the overall group analysis. The direct effects of depressive symptoms, cognitive efficiency, and sleep disturbances on mistakes at work were all significant at *p* < 0.001.

The direct effect of sleep disturbances was 0.35, the most influential value among other variables. The direct effect of cognitive efficiency and depressive symptoms were 0.18, and 0.08, respectively. The relationships of depressive symptoms and sleep disturbances with mistakes at work were both significantly mediated by cognitive efficiency (*p* < 0.001) (Supplementary Table S2).

**Figure 2:**
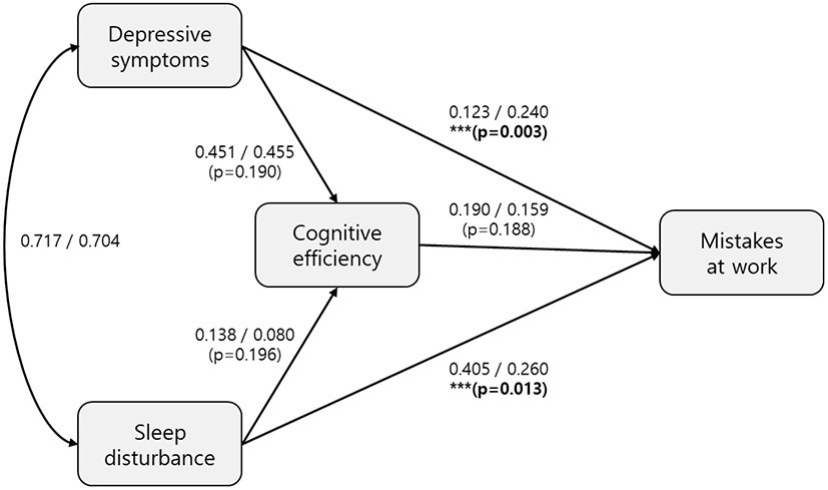
Structural equation model of the effects of depressive symptoms, sleep disturbances and cognitive efficiency on mistakes at work by shift and non-shift workers. The structural equation model includes sleep disturbances, depressive symptoms, cognitive efficiency, and mistakes at work by shift and non-shift workers. The path coefficients for the shift and non-shift workers are on the left and right side of the slashes, respectively. The models for shift and non-shift workers both fit the data well: χ^2^ (df = 114, *n* = 4,561) = 2,812.582, *p* < 0.001, CFI = 0.933, TLI = 0.910, RMSEA = 0.072, 95% CI RMSEA [0.070, 0.074] for shift workers and χ^2^ (df = 114, *n* = 2,093) = 1,153.725, p <0.001, CFI = 0.937, TLI = 0.916, RMSEA = 0.066, 95% CI RMSEA (0.063, 0.070) for non-shift workers. In the shift workers, sleep disturbances were associated with cognitive efficiency (path coefficient = 0.138; *p* < 0.001) and mistakes at work (path coefficient = 0.405; *p* < 0.001), while depressive symptoms were not significantly associated with mistakes at work (path coefficient = 0.123; *p* = 0.193). In the non-shift workers, sleep disturbances were associated with cognitive efficiency (path coefficient = 0.080; *p* = 0.035) and mistakes at work (path coefficient = 0.260; *p* < 0.001), while depressive symptoms were associated with mistakes at work (path coefficient = 0.240; *p* < 0.001). The path coefficient between cognitive efficiency and mistakes at work was statistically significant (*p* < 0.001) in both shift and non-shift workers. The comparison of path coefficients between shift and non-shift workers were tested using multi-group structural equation model. The *p*-values of path coefficient comparisons were presented in (). ***Indicates a statistically significant difference between shift and non-shift workers.

The individual models for shift and non-shift workers fit the data well [χ^2^ (df = 114, *n* = 4,561) = 2,812.582, *p* < 0.001, CFI = 0.933, TLI = 0.910, RMSEA = 0.072, 95% CI RMSEA (0.070, 0.074) for the shift workers and χ^2^ (df = 114, *n* = 2,093) = 1,153.725, *p* < 0.001, CFI = 0.937, TLI = 0.916, RMSEA = 0.066, 95% CI RMSEA (0.063, 0.070) for the non-shift workers]. For the shift workers, sleep disturbances was associated with cognitive efficiency (path coefficient = 0.138; *p* < 0.001) and mistakes at work (path coefficient = 0.405; *p* < 0.001), but depressive symptoms was not significantly associated with mistakes at work (path coefficient = 0.123; *p* = 0.193). For the non-shift workers, sleep disturbances were associated with cognitive efficiency (path coefficient = 0.080; *p* = 0.035) and mistakes at work (path coefficient = 0.260; *p* < 0.001); depressive symptoms was also associated with mistakes at work (path coefficient = 0.240; *p* < 0.001). All other path coefficients were statistically significant at *p* < 0.001 (Figure 2).

The multi-group SEM analyses showed a significant difference in the magnitude of path coefficients between the two groups (Figure 2). The path coefficients of sleep disturbances and depressive symptoms on mistakes at work were significantly different between the groups (*p* = 0.013 and 0.003, respectively).

## Discussion

The current study used an SEM to investigate the influence of depressive symptoms, sleep disturbances, and cognitive efficiency on mistakes at work. We aimed to improve understanding of the differences in these pathways between shift and non-shift workers. To the best of our knowledge, this study was the first to explore the direct and indirect effects of these interrelated variables on mistakes at work in shift and non-shift workers.

Consistent with our hypothesis, shift workers made more mistakes at work compared to non-shift workers. Shift work was also associated with more depressive symptoms, poorer sleep quality, and lower cognitive efficiency. As expected, depression, sleep, cognition, and performance were closely interrelated, both in shift and non-shift workers. Moreover, our findings showed that depression, sleep, and cognition had significant effects on mistakes at work in both shift and non-shift workers. As mistakes at work were positively correlated with depression, sleep, and cognition in both groups, the greater frequency of mistakes in shift workers may be due to more severe depressive symptoms, and poorer cognitive efficiency and sleep.

Depression, sleep, and cognition influenced work performance in overall groups, and cognition mediated the well-established association between sleep and performance. This finding suggests that the role of cognition is important to understand how sleep affects performance at work. Our results were in line with previous studies reporting that cognitive efficiency mediates the relationship between sleep and performance in both academic and occupational settings (28). Global cognitive processes, including not only basic attention/sustained vigilance (14), but also higher executive functioning (29), are vulnerable to be affected by sleep deprivation. Executive function deficits may cause sluggishness, tiredness/lethargy, and slowed thinking or processing, which leads to impaired performance (30). A mediating effect of cognition on the relationship between sleep and performance was found in both shift and non-shift workers, suggesting that cognition may be crucial to performance regardless of working conditions.

The major finding of our study was that there was a group difference in pathways to mistakes in performance. In shift workers, cognitive efficiency mediated the relationship between sleep and performance, and there was no significant effect of depression on performance. On the other hand, all of the tested pathways involving cognitive efficiency were significant in non-shift workers. One possible explanation for this is that depression in shift workers may be significantly affected by sleep disturbances. Moderate to severe sleep disturbances were common in the shift workers in the current study, while depressive symptoms did not reach clinical or subclinical levels in most of those workers. This suggests that sleep problems might be the main factor impairing cognitive functioning, leading to mistakes at work by shift workers. In addition, depressive symptoms in shift workers may be mild or independent from the sleep disturbances.

The multi-group analysis showed that the overall effect of sleep on performance was greater in shift workers than non-shift workers, as the overall effect of depression on performance was greater in non-shift workers. In other words, sleep had a greater impact on performance in shift workers, while mood had a greater impact on performance in non-shift workers. Shift workers may experience a greater physical burden with diverse health problems due to the working conditions. Even shift workers marginally adapted to the conditions can experience long-term sleep disturbances, which may reduce tolerance and resilience (31–33). In this case, even a slight change in sleep pattern may have a large impact on cognition and performance in shift workers. Whereas, shift workers are primarily vulnerable to sleep disturbances, non-shift workers might be affected by many factors other than sleep. For example, mood problems due to work-related stress, interpersonal conflict, or burnout might disturb the performance of employees. Thus, intervention to regulate mood and manage stress might be helpful for non-shift workers.

The importance of sleep on performance in shift workers in the current study indicates the necessity of sleep-targeted interventions. For example, cognitive behavioral therapy for insomnia could improve performance in shift workers. In cases where flexible work schedules prevent face-to-face interventions, digital or internet-based therapies may be good alternatives, especially for shift workers.

The main strengths of this study included the use of a multidimensional model, which integrated multiple factors that may influence performance, and the fact that it was the first study to use a multi-group SEM to compare the effects of sleep, depression, and cognition on performance between shift and non-shift workers. In addition, the study had a large sample size collected through the online survey and well-validated instruments were used to evaluate sleep, depression, and cognition, which increased the reliability and validity of the results.

The study also had some methodological limitations. First, there was a potential selection bias. As all of the respondents participated in the survey voluntarily, workers with severer psychopathologies might have been excluded. Second, as performance was assessed using only two items, various aspects of performance other than mistakes may have been overlooked. Third, self-reported questionnaires were used instead of objective measures of sleep (e.g., actigraphy and polysomnography) and work performance (e.g., labor productivity). Objective evaluation of these factors may yield more understanding in future studies.

## Conclusion

The current study demonstrated close associations of sleep, depression, and cognition, with work performance. Cognition mediated the relationship between sleep and performance in both shift and non-shift workers. This study provides insight into the causal relationship between sleep and performance with mediating role of cognition. Notably, sleep disturbance was an important factor with respect to mistakes at work, especially by shift workers. Sleep should be considered as a factor that affects functioning in shift workers, both independently and in association with other factors.

Even when other work-related factors were taken into account, sleep problems may be the main cause of performance impairments in night-time or rotating or irregular shift workers. Non-shift workers can maintain their sleep-wake pattern constant, but their performances may also be compromised by work-related stressors other than the sleep-wake cycle. Therefore, it is necessary to provide individual interventions for employees to well-function in the work system.

## Data availability statement

The original contributions presented in the study are included in the article/Supplementary material, further inquiries can be directed to the corresponding author.

## Ethics statement

The studies involving human participants were reviewed and approved by the Institutional Review Board of Samsung Medical Center (Protocol Code: 2019-04-095). The patients/participants provided their written informed consent to participate in this study.

## Author contributions

The data collection of the project was done by JL, SL, and JK. The idea for the paper, the data analysis, and the writing was done by HY. The data cleaning is done by YH. The review of the paper and suggested ideas were done by SJ ad SK. The review and final edits of the paper were done by SK. All authors contributed to the article and approved the submitted version.
