# Supplementary material for: Sleep disturbances, depressive symptoms, and cognitive efficiency as determinants of mistakes at work in shift and non-shift workers
Source: Front Public Health. 2022 Dec 14;10:1030710. doi: 10.3389/fpubh.2022.1030710 (PMC9794856; doi:10.3389/fpubh.2022.1030710)
Supplement: Supplementary file 1 [file Data_Sheet_1.PDF]

Supplementary Material

Supplementary Table S1. Spearman correlation analysis between PSQI, CFA, CES-D, and mistakes at work by all participants

	PSQI	CFA	CES-D	Mistakes at work
PSQI	1			
CFA	.342***	1		
CES-D	.509***	.548***	1	
Mistakes at work	.308***	.358***	.353***	1

PSQI, Pittsburg Sleep Quality Index; CES-D, Center for Epidemiologic Studies Depression Scale; CFQ, Cognitive Failure Questionnaire.

$^{***}p < 0.01$ in Spearman's correlation analysis.

Supplementary Table S2. Effect decomposition of depressive symptoms, sleep disturbances, and cognitive efficiency on mistakes at work in all participants, shift workers, and non-shift workers: results of the structural equation model

Group	Predictor	Response	Direct effect	Indirect effect	Overall effect	P-value
Overall						
	Sleep	Cognition	0.113	-	0.113	< 0.001
	Depression	Cognition	0.462	-	0.462	< 0.001
	Sleep	Performance	0.350	0.020	0.370	< 0.001
	Depression	Performance	0.078	0.084	0.162	0.001
	Cognition	Performance	0.181	-	0.181	< 0.001
Shift workers						
	Sleep	Cognition	0.138	-	0.138	< 0.001
	Depression	Cognition	0.451	-	0.451	< 0.001
	Sleep	Performance	0.379	0.026	0.405	< 0.001
	**Depression**	**Performance**	**0.038**	**0.086**	**0.123**	**0.193**
	Cognition	Performance	0.190	-	0.190	< 0.001
Non-shift workers						
	Sleep	Cognition	0.080	-	0.080	0.035
	Depression	Cognition	0.455	-	0.455	< 0.001
	Sleep	Performance	0.252	0.013	0.260	< 0.001
	Depression	Performance	0.168	0.073	0.240	< 0.001
	Cognition	Performance	0.159	-	0.159	< 0.001

The overall effect of all variables (except for depressive symptoms) on performance was significant in shift workers ($p < 0.001$). All path coefficients were significant at $p < 0.05$ in the overall and non-shift worker groups.

Sleep, sleep disturbance; Cognition, cognitive efficiency; Depression, depressive symptoms; Performance, mistakes at work.

Supplementary Figure S1. Structural equation model (SEM) of the effects of depressive symptoms, sleep disturbances and cognitive efficiency, on mistakes at work by all participants.

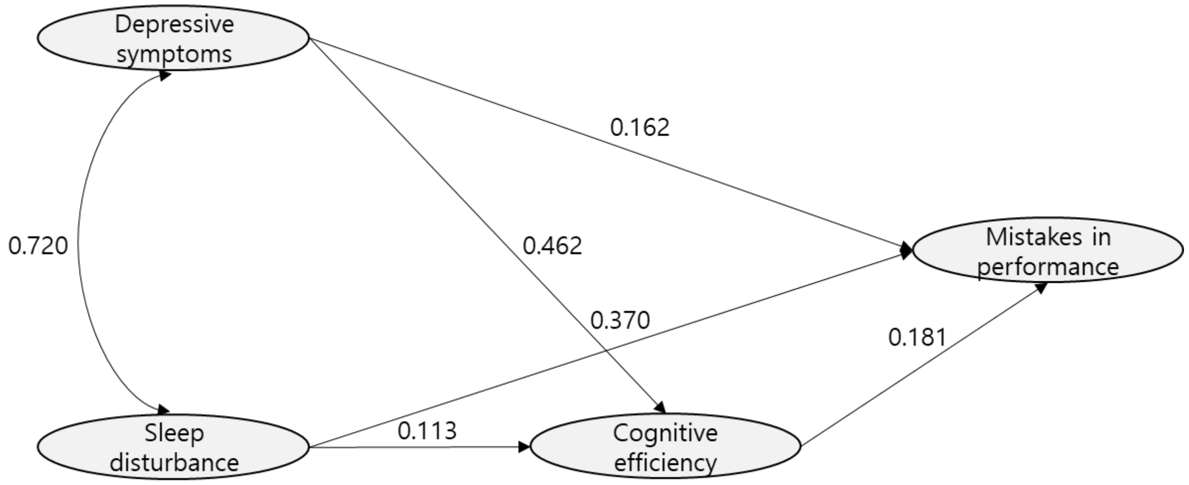

SEM goodness of fit: chi square = 3,874.272, df = 114, CFI = 0.935, TLI = 0.912, RMSEA = 0.070. The overall effect and sums of the direct and indirect effects of all hypothesized pathways were statistically significant ($p < 0.001$). Cognitive efficiency mediated the effects of depressive symptoms and sleep disturbances on mistakes at work.

Supplementary Figure S2. Multi-group structural equation model (SEM) of the effects of depressive symptoms, sleep disturbances, and cognitive efficiency on mistakes at work by shift workers.

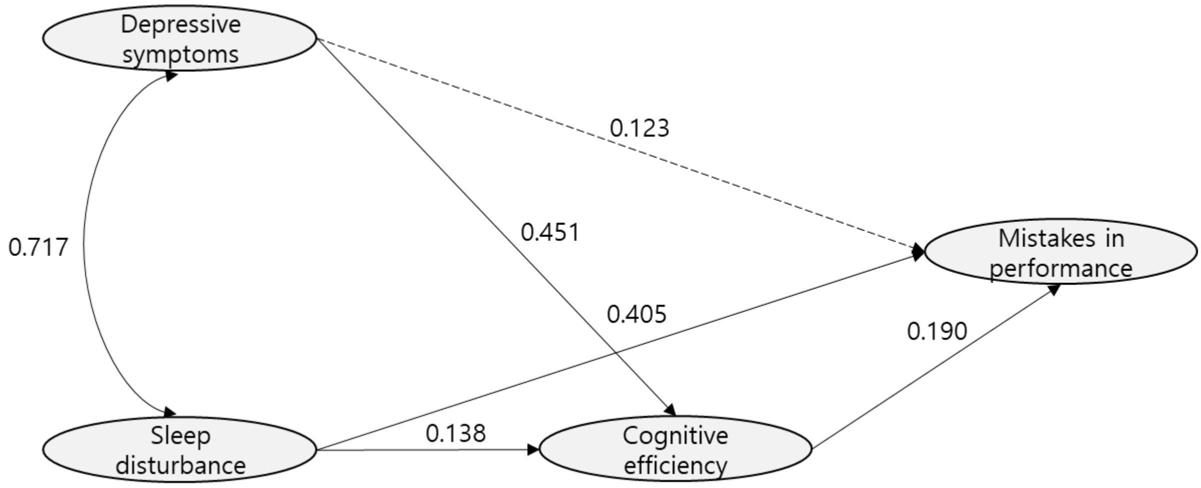

Multi-group SEM goodness of fit: chi square = 3,966.283, df =228, CFI = 0.934, TLI = 0.912, RMSEA = 0.050. The direct and indirect effects of depressive symptoms on mistakes at work were not significant ($p = 0.193$). All other pathways were significant, and cognitive efficiency mediated the effects of sleep disturbances on mistakes at work ($p < 0.001$).

Supplementary Figure S3. Multi-group structural equation model (SEM) of the effects of depressive symptoms, sleep disturbances, and cognitive efficiency on mistakes at work by non-shift workers.

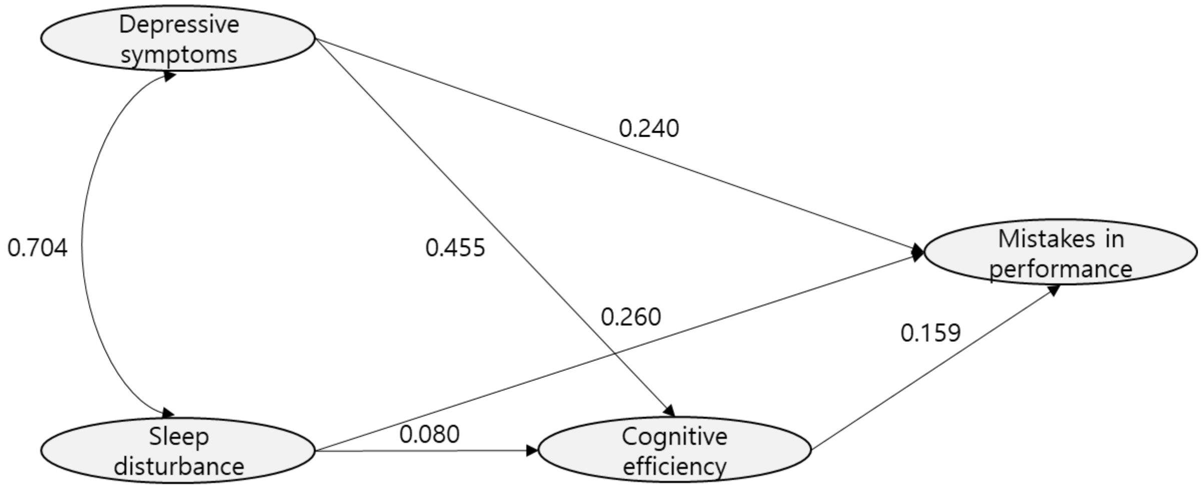

The overall effects of all hypothesized pathways were statistically significant ($p < 0.001$). Cognitive efficiency mediated the effects of both depressive symptoms and sleep disturbances on mistakes at work.

SPSS Statistics (version 27.0; IBM Corp., Armonk, NY, USA) was used for confirmatory factor analysis (CFA) and exploratory factor analysis (EFA), which were performed to examine the structure and psychometric properties of each scale.

Previous studies suggested a 7-factor model for the K-PSQI (Shin & Kim, 2020), 4-factor model for the K-CES-D (Hoe, Park & Bae, 2015) and 5-factor model for the K-CFQ (Park & Kang, 2011). CFA was performed to ascertain the replicability of these factor structures in our participants, while EFA was used to explore the construct validity of the two items assessing mistakes at work, due to a lack of evidence in literature.

To evaluate model fit, the Tucker-Lewis Index (TLI) and comparative fit index (CFI), absolute model fit indices such as the chi-square ($\chi 2$), root mean square error of approximation (RMSEA), and standardized root mean squared residual (SRMR) statistics were generated. The Akaike's information criterion (AIC) was applied to find the applicability of factor analysis. Model fit was considered good when the CFI and TLI were > 0.95, RMSEA was < 0.08 (Bentler, 1990), and SRMR was ≤ 0.08 (Hu & Bentler, 1999). Also, the lower the AIC, the better the model fit (Akaike, 1987).

All models showed good model fits. A summary of the goodness-of-fit indices for the CFA is presented in Supplementary Table S3. The factor model and its factor loadings are depicted in Supplementary Figure S4. The one-factor model was derived from EFA, which also showed a good fit according to all indices.

Supplementary Table S3. Summary of goodness-of-fit indices for CFA

Model tested	χ2	AIC	CFI	TLI	RMSEA	90% CI
7-factor model for K-PSQI	872.62*** (df = 14)	914.62	0.890	0.781	0.096	0.091–0.101
4-factor model for K-CES-D	577.22*** (df = 29)	649.22	0.984	0.975	0.053	0.050–0.057
5-factor model for K-CFQ	12851.40*** (df = 220)	13009.40	0.873	0.854	0.093	0.091–0.094

AIC, Akaike information criterion; CFI, comparative fit index; TLI, Tucker-Lewis Index; RMSEA, root mean square error of approximation; CI, confidence interval. ***p < 0.001.

Supplementary Figure S4. Confirmatory factor analysis of the 7-factor model for K-PSQI, 4-fator model for K-CES-D, and 5-factor model for K-CFQ.

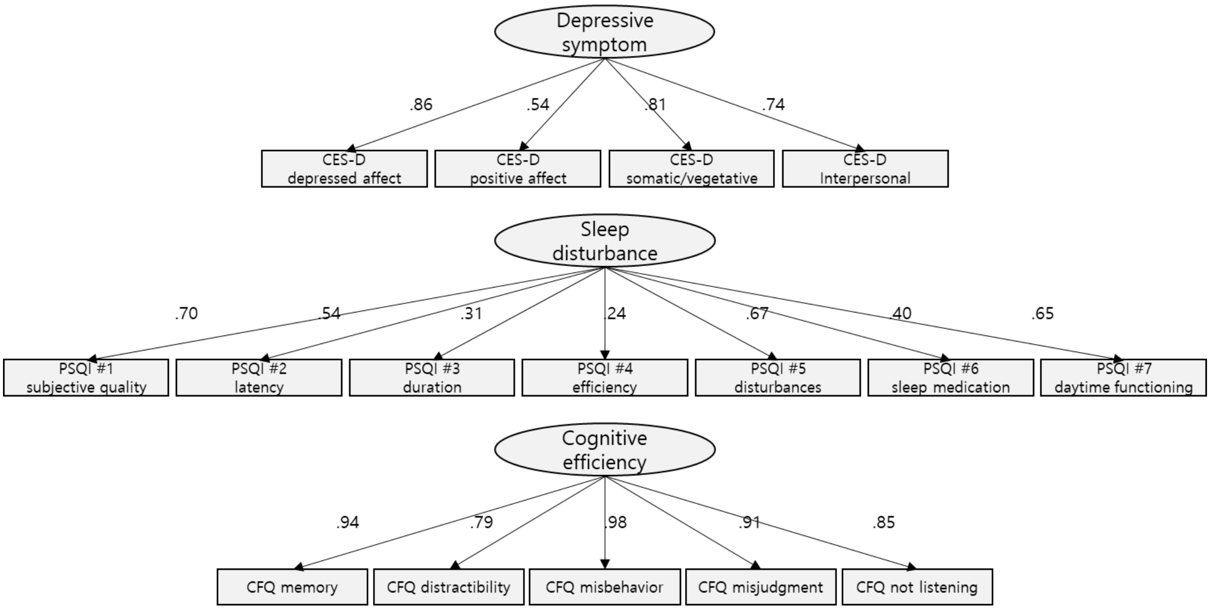

All coefficients are significant at $p < 0.001$.

Reference

Akaike, H. (1987). Factor Analysis and AIC. *Psychometrika* 371–386.

Bentler, P. M. (1990). Comparative fit indexes in structural models. *Psychological Bulletin*. 107, 238–246. https://doi.org/10.1037/0033-2909.107.2.238.

Park, C. H., & Kang, H. (2011). [A study on the validation of cognitive failure questionnaire: case of Korean college students]. *Korean Journal of Psychology*, 30, 341-55.

Shin, S., & Kim, S. H. (2020). The Reliability and Validity Testing of Korean Version of the Pittsburgh Sleep Quality Index. *Journal of Convergence for Information Technology*, 10(11), 148-155. https://doi.org/10.1007/s11325-011-0579-9

Hoe, M. S., Park, B. S., & Bae, S. W. (2015). [Testing measurement invariance of the 11-item Korean version CES-D scale]. *Mental Health and Social Work*, 43(2), 313-339.

Hu, L. T., & Bentler, P. M. (1999). Cutoff criteria for fit indexes in covariance structure analysis: conventional criteria versus new alternatives. *Structural Equation Modeling*, 6, 1–55. https://doi.org/10.1080/10705519909540118
